# SUMOylation Regulator-Related Molecules Can Be Used as Prognostic Biomarkers for Glioblastoma

**DOI:** 10.3389/fcell.2021.658856

**Published:** 2021-04-09

**Authors:** Xiaozhi Li, Yutong Meng

**Affiliations:** ^1^Department of Neurosurgery, Shengjing Hospital of China Medical University, Shenyang, China; ^2^Department of Stomatology, Shengjing Hospital of China Medical University, Shenyang, China

**Keywords:** glioblastoma, prognosis, SUMOylation, post-translational modification, risk assessment model

## Abstract

**Introduction:**

SUMOylation is one of the post-translational modifications. The relationship between the expression of SUMOylation regulators and the prognosis of glioblastoma is not quite clear.

**Materials and Methods:**

The single nucleotide variant data, the transcriptome data, and survival information were acquired from The Cancer Genome Atlas, Gene Expression Omnibus, and cBioportal database. Wilcoxon test was used to analyze differentially expressed genes between glioblastoma and normal brain tissues. Gene set enrichment analysis was conducted to find the possible functions. One risk scoring model was built by the least absolute shrinkage and selection operator Cox regression. Kaplain–Meier survival curves and receiver operating characteristic curves were applied to evaluate the effectiveness of the model in predicting the prognosis of glioblastoma.

**Results:**

Single-nucleotide variant mutations were found in SENP7, SENP3, SENP5, PIAS3, RANBP2, USPL1, SENP1, PIAS2, SENP2, and PIAS1. Moreover, UBE2I, UBA2, PIAS3, and SENP1 were highly expressed in glioblastoma, whereas PIAS1, RANBP2, SENP5, and SENP2 were downregulated in glioblastoma. Functional enrichment analysis showed that the SUMOylation regulators of glioblastoma might involve cell cycle, DNA replication, and other functions. A prognostic model of glioblastoma was constructed based on SUMOylation regulator-related molecules (ATF7IP, CCNB1IP1, and LBH). Kaplain–Meier survival curves and receiver operating characteristic curves showed that the model had a strong ability to predict the overall survival of glioblastoma.

**Conclusion:**

This study analyzed the expression of 15 SUMOylation regulators in glioblastoma. The risk assessment model was constructed based on the SUMOylation regulator-related genes, which had a strong predictive ability for the overall survival of patients with glioblastoma. It might provide targets for the study of the relationship between SUMOylation and glioblastoma.

## Introduction

Glioblastoma is the most malignant intracranial tumor originating from glial cells. Common treatments for glioblastoma include microsurgery, the use of chemotherapy drugs, such as temozolomide, and radiotherapy. However, the prognosis of patients with glioblastoma is still very poor. Studies have shown that the 5-year survival rate is only approximately 5% (Stupp et al., 2009; Ostrom et al., 2017). Due to the high cost of treatment of glioblastoma and poor treatment effect, it has always been one of the difficulties in tumor treatment (Cloughesy et al., 2020; Faria et al., 2020; Reardon et al., 2020). Therefore, it is of great importance to find effective molecular targets and improve the prognosis of glioblastoma.

Epigenetic modification is a heritable change in gene expression without DNA change, which plays a very important role in the development of glioblastoma (Kelly and Issa, 2017; Uddin et al., 2020). SUMOylation is one of the post-translational modifications. Through the catalysis of tertiary enzymes, the small ubiquitin-like modifier (SUMO) proteins bind to lysine residues (Rodriguez et al., 2001; Sampson et al., 2001), which can play various regulatory roles in tumor subcellular localization and transcription activities (Eifler and Vertegaal, 2015; Seeler and Dejean, 2017). The process of SUMOylation requires the activation of three kinds of enzymes (Johnson, 2004). SUMO-activating enzymes (E1, including SAE1, UBA2) can form heterodimers and transfers SUMO molecules to SUMO-conjugating enzyme (E2, including UBE2I). The SUMO-conjugating enzyme then transfers SUMO molecules to the lysine residue of the substrate. SUMO ligases (E3, including PIAS1, PIAS2, PIAS3, PIAS4, and RANBP2) make the substrate bind more closely to SUMO molecules. In addition, SUMOylation can be reversed by SUMO proteases (SENP1, SENP2, SENP3, SENP5, SENP6, SENP7, and USPL1), which is also called deSUMOylation. However, the relationship between the expression of SUMOylation regulators and the prognosis of glioblastoma is not quite clear.

This study aims to analyze the genome and transcriptome of glioblastoma and explore the expression characteristics and prognostic value of SUMOylation regulators and their related molecules in glioblastoma.

## Materials and Methods

### Data Sources

The single-nucleotide variant (SNV) data of glioblastoma came from The Cancer Genome Atlas (TCGA) database^1^. The transcriptome data of glioblastoma was acquired from the TCGA database and the Gene Expression Omnibus (GEO) database (GSE13041 and GSE83300)^2^. The survival information of the TCGA dataset came from the cBioportal database^3^.

### Data Processing

Based on the data acquired, the SNV of SUMOylation regulators of glioblastoma was analyzed by the R language “maftools” package. The intersection genes of TCGA and GEO datasets were taken for subsequent expression and prognostic analysis. Wilcoxon test was used to analyze differentially expressed genes between glioblastoma and normal brain tissues. A heatmap was drawn for visualization. Because isocitrate dehydrogenase (IDH) mutations have a great impact on the prognosis of glioblastoma, we further analyzed the expression of SUMOylation regulators in different IDH mutation subgroups.

### Functional Enrichment Analysis

To explore the biological functions that SUMOylation might regulate, the R language “clusterProfiler” package performed Kyoto Encyclopedia of Genes and Genomes (KEGG) enrichment analysis of glioblastoma genes.

### Construction of a Prognostic Model

To find out the prognostic importance of SUMOylation regulator-related molecules, a prognostic model of glioblastoma was constructed. The TCGA dataset was used as the training group, whereas the GEO datasets (GSE13041 and GSE83300) were used as the validation group. In the training group, the differentially expressed genes between glioblastoma and normal brain tissue were identified first. When the Pearson correlation coefficient was greater than 0.6, related molecules of the SUMOylation regulators were selected. Correlations between genes and overall survival of glioblastoma were performed by univariate Cox regression. Later, a risk-scoring model was built by the least absolute shrinkage and selection operator Cox regression. Kaplain–Meier survival curves and ROC curves were drawn in both the training group and the validation group to evaluate the effectiveness of the model in predicting the prognosis of glioblastoma. The nomogram was presented accordingly.

### Statistical Analysis

R language (4.0.2) was used for data analysis and graphing. When *P* < 0.05, it was considered statistically significant.

## Results

### Single-Nucleotide Variant Overview of SUMOylation Regulators in Glioblastoma

**FIGURE 1:**
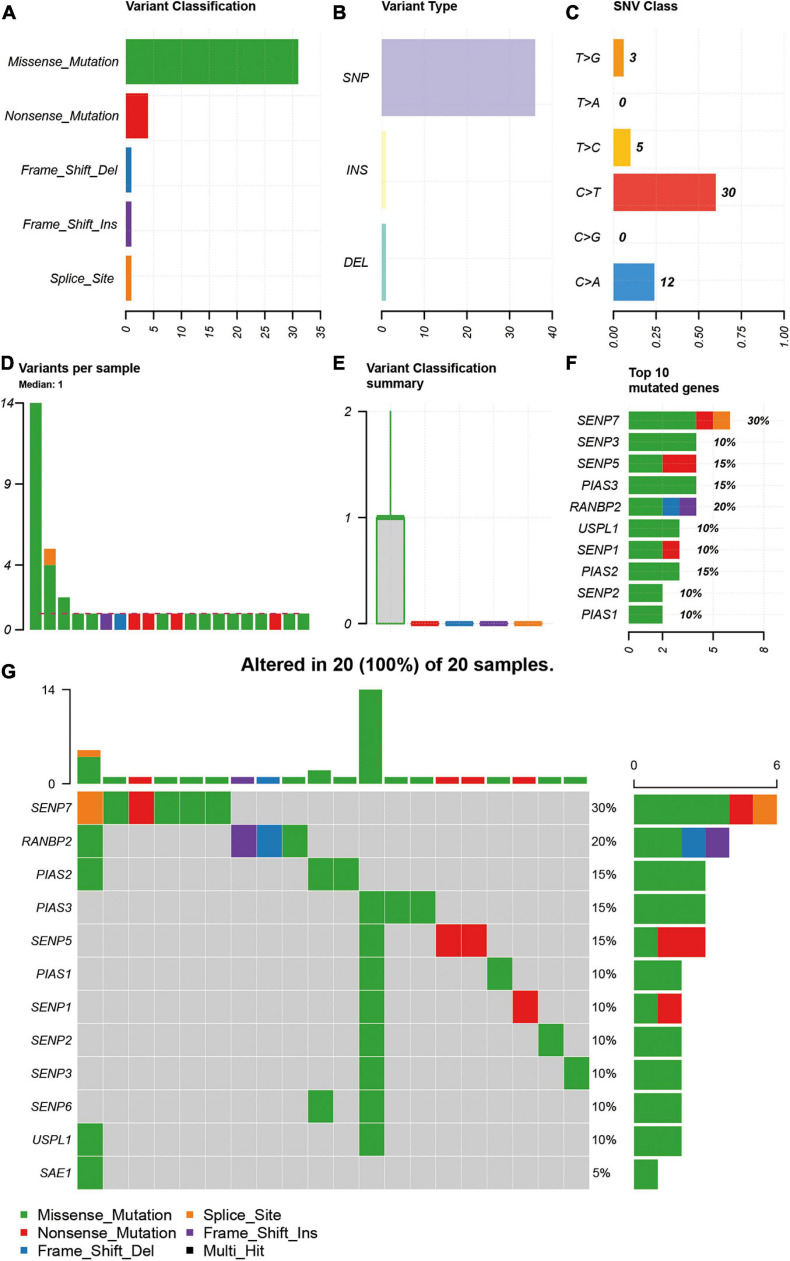
Description of SNV of SUMOylation regulators in glioblastoma. **(A–C)** Missense mutation, SNP, and C > T were the main SNV types. **(D,E)** Most cases only have one single SNV of SUMOylation regulators. **(F)** SENP7 had the highest SNV frequency. **(G)** Relationship between the distribution of the SNV and the cases.

As SNV status was often associated with abnormal gene expression, we first checked the SNV status of SUMOylation regulators. From the perspective of SNV types, missense mutation, SNP, and C > T were the main mutation forms, as shown in Figures 1A–C. Most cases only have one single SNV of SUMOylation regulators (Figures 1D,E). SENP7, SENP3, SENP5, PIAS3, RANBP2, USPL1, SENP1, PIAS2, SENP2, and PIAS1 showed SNV, and SENP7 had the highest SNV frequency (Figure 1F). The relationship between the distribution of the SNV and the cases are shown in Figure 1G. Because of the low SNV frequency of SUMOylation regulators (20/590), we could not accurately estimate the exact relationship between SNV and certain clinical characteristics, which required more sequencing studies by researchers.

### Differentially Expressed SUMOylation Regulators

**FIGURE 2:**
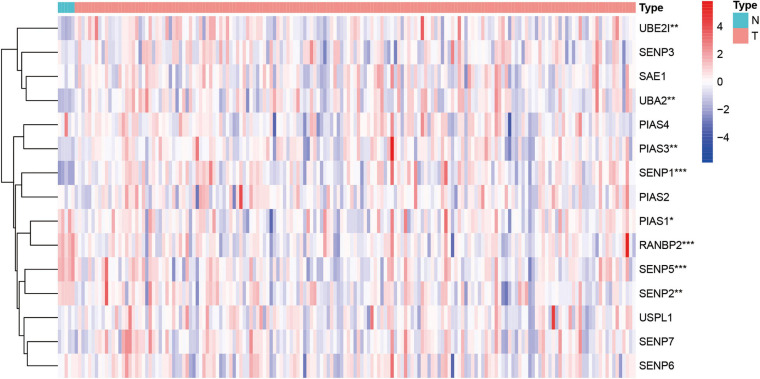
Heatmap of all SUMOylation regulators in glioblastoma. *p < 0.05, **p < 0.01, and ***p < 0.001.

**FIGURE 3:**
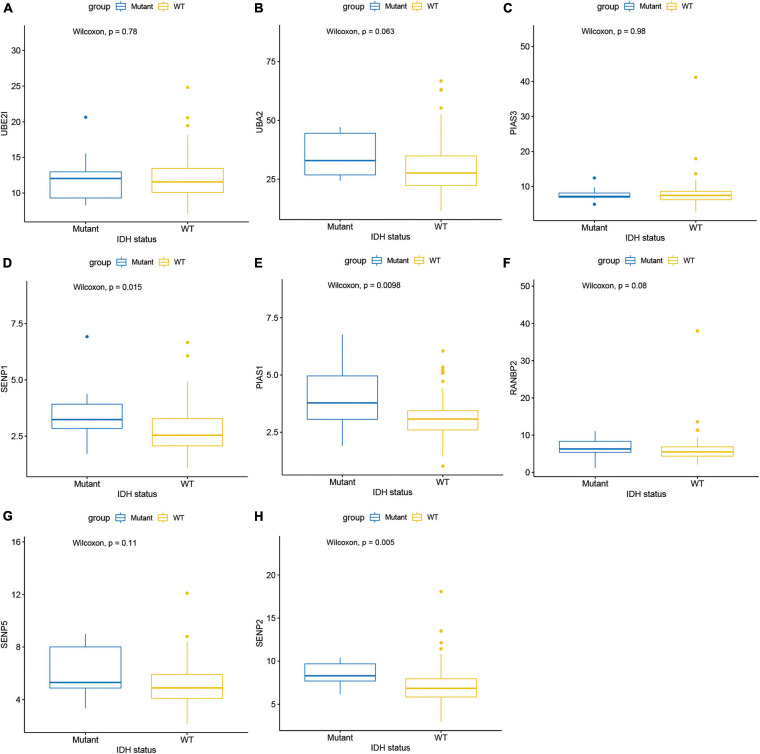
Expression in different IDH mutation status subgroups of **(A)** UBE2I, **(B)** UBA2, **(C)** PIAS3, **(D)** SENP1, **(E)** PIAS1, **(F)** RANBP2, **(G)** SENP5, and **(H)** SENP2.

Next, this study investigated the expression difference of SUMOylation regulators between glioblastoma and normal brain tissue. The heatmap of the expression of all SUMOylation regulators in glioblastoma is shown in Figure 2. As can be seen from the figure, UBE2I, UBA2, PIAS3, and SENP1 were upregulated in glioblastoma, whereas PIAS1, RANBP2, SENP5, and SENP2 were downregulated in glioblastoma. Among the eight differentially expressed genes, SENP1, PIAS1, and SENP2 also had different expressions in different IDH mutation status subgroups. Compared with the IDH mutant type, these three genes were downregulated in the IDH wild type, as shown in Figure 3. Although many SUMOylation regulators were abnormally expressed in glioblastoma or in different glioblastoma IDH status, we did not find their significant predictive effect on overall survival. We presume this might be because SUMOylation was not the original driving factor of glioblastoma or might play a role at a certain stage of glioblastoma development. This phenomenon required further research into SUMOylation in the future.

### Functional Enrichment Analysis

**FIGURE 4:**
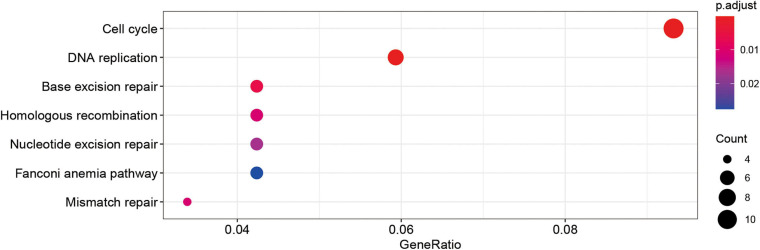
Kegg analyses showed the SUMOylation regulators might be related to cell cycle, DNA replication, and other functions.

By calculating the correlation between genes, the upstream and downstream genes of the target gene can be found to gain a deeper understanding of the biological functions of the target gene. In this study, KEGG enrichment analysis was performed on genes whose correlation coefficients with SUMOylation regulators were greater than 0.6. The results are shown in Figure 4. SUMOylation regulators may be related to cell cycle, DNA replication, and other functions. These results suggested that SUMOylation might be involved in the regulation of glioblastoma cell proliferation.

### Construction of a Glioblastoma Prognostic Model Based on SUMOylation Regulator-Related Molecules

**FIGURE 5:**
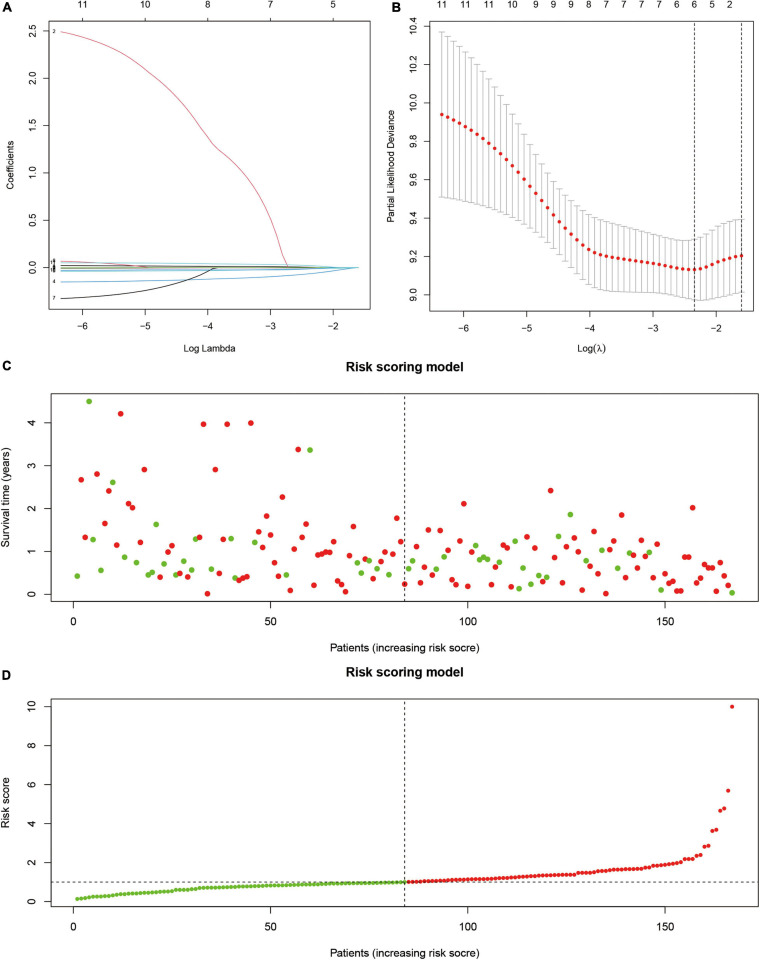
Prognostic model based on SUMOylation regulator-related genes. **(A)** Lambda selection in the least absolute shrinkage and selection operator model. **(B)** Least absolute shrinkage and selection operator coefficient profiles of selected genes. **(C,D)** Relationship between overall survival and risk score distribution.

Next, we used survival analysis to identify the SUMOylation-related prognostic signatures and test their prognostic values. Two hundred thirty-nine SUMOylation regulator-related molecules were differentially expressed in glioblastoma (|log 2 FC| > 1). Univariate Cox regression results suggested that KANK2, MYO15A, SEMA3F, ATF7IP, CCNB1IP1, HNRNPC, PTGIR, ZNF85, PXDN, ZNF432, and LBH were closely related to the prognosis of glioblastoma. The risk-scoring model for glioblastoma was constructed by the least absolute shrinkage and selection operator Cox regression (Figures 5A,B). The risk score was expressed by the following equation: Risk score = –0.138 × Expression ATF7IP-0.047 × Expression CCNB1IP1 + 0.054 × Expression LBH. The relationship between overall survival and risk score distribution is shown in Figures 5C,D.

**FIGURE 6:**
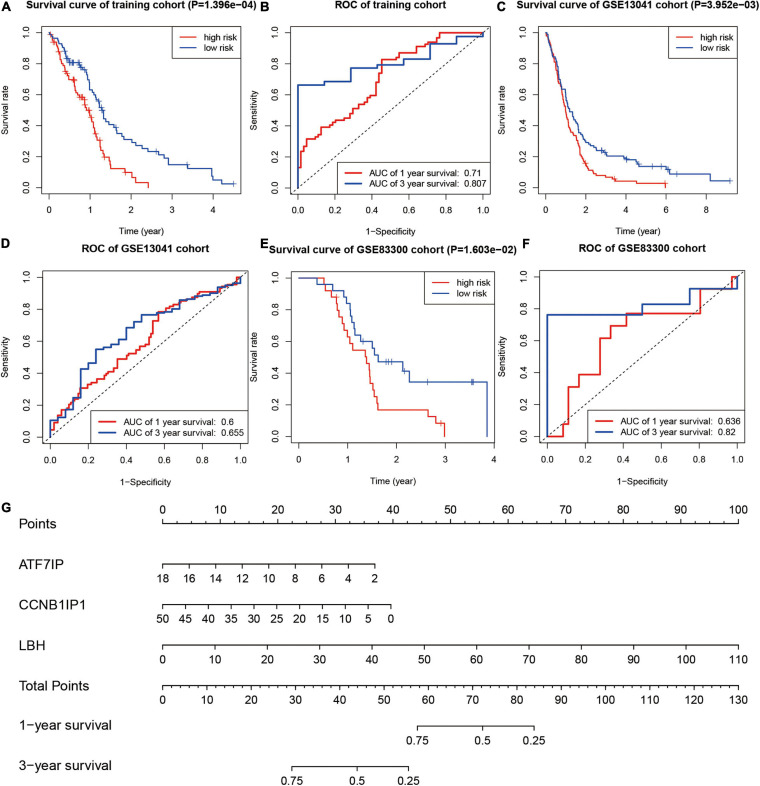
Testing of the prognostic model. **(A,B)** Kaplan–Meier survival curve and ROC curve of the training group. **(C,D)** Kaplan–Meier survival curve and ROC curve of the GSE13041 group. **(E,F)** Kaplan–Meier survival curve and ROC curve of the GSE83300 group. **(G)** Nomogram for predicting the overall survival of glioblastoma patients.

In the training group, divided by the median risk score, the overall survival of patients with high-risk scores was significantly worse than that of patients with low-risk scores (Figure 6A, *P* < 0.01). The 1- and 3-year areas under the curve of ROC curves of the training group were 0.710 and 0.807, respectively (Figure 6B). In one validation group (GSE13041), the overall survival of patients with high-risk scores was also significantly worse than that of patients with low-risk scores (Figure 6C, *P* < 0.01). The 1- and 3-year areas under the curve of ROC curves in the GSE13041 group were 0.600 and 0.655, respectively (Figure 6D). In the other validation group (GSE83300), the survival curve and ROC plot also showed the predictive power of the model, as shown in Figures 6E,F. These results showed that the risk-scoring model based on SUMOylation-related signatures performed well in predicting the overall survival of glioblastoma patients. Finally, a nomogram based on the risk scoring model is drawn in Figure 6G.

## Discussion

This study explored the characteristics of SUMOylation in glioblastoma in terms of SNV, gene expression, functional enrichment, and prognostic values. In this study, we first analyzed the SNV of 15 SUMOylation regulators. SNV mutations were found in SENP7, SENP3, SENP5, PIAS3, RANBP2, USPL1, SENP1, PIAS2, SENP2, and PIAS1. Next, we analyzed the expression of all the SUMOylation regulators in glioblastoma. UBE2I, UBA2, PIAS3, and SENP1 were highly expressed in glioblastoma. PIAS1, RANBP2, SENP5, and SENP2 were downregulated in glioblastoma. However, the SNV frequency of SUMOylation regulators in glioblastoma was relatively low compared with the well-known IDH. Then, KEGG enrichment analysis showed that the SUMOylation regulators of glioblastoma might involve cell cycle, DNA replication, and other functions. Finally, a prognostic model of glioblastoma was constructed based on SUMOylation regulator-related molecules (ATF7IP, CCNB1IP1, and LBH). Kaplain–Meier survival curves and ROC curves showed that the model had a strong ability to predict the overall survival of glioblastoma.

SUMOylation was first discovered in the 1990s (Matunis et al., 1996; Mahajan et al., 1997). It regulates the localization and activity of a variety of proteins (Yeh et al., 2000; Geiss-Friedlander and Melchior, 2007). The unbalanced regulation of SUMOylation and deSUMOylation is one of tumor pathogenesis (Wu et al., 2020, 2021; Samaržija, 2021). UBA2 is highly expressed in small cell lung cancer. Silencing UBA2 can reduce tumor cell migration and invasion ability and increase the sensitivity to etoposide and cisplatin (Liu et al., 2015). UBE2I is the only SUMO conjugating enzyme that is highly expressed in colon cancer and prostate cancer, whereas it is downregulated in breast, prostate, and lung cancers (Moschos et al., 2010). In breast cancer, overexpressed SENP2 enhances the deSUMOylation of NEMO and inhibits the activation of nuclear factor kappa-light-chain-enhancer of activated B cells (Gao et al., 2019). In addition, PIAS3 is upregulated in lung cancer, breast cancer, prostate cancer, colorectal cancer, and brain tumors (Wang and Banerjee, 2004). The SUMOylation pathway and drugs targeting SUMOylation are promising to provide new strategies for tumor treatment (He et al., 2017; Yang et al., 2018).

So far, there are limited treatment options for glioblastoma, and even with the existing standard treatment, the overall survival and quality of life of patients are still very poor. Therefore, the research on the pathogenesis of glioblastoma and the corresponding molecular targeted drugs are quite promising. Topotecan, a United States Food and Drug Administration-approved anti-glioblastoma drug, can reduce overall cell SUMOylation, CDK6, and HIF-1α levels and regulate the cell cycle, but the specific target is not clear (Zheng et al., 2020).

As one of the important results of this study, we found that UBE2I, UBA2, PIAS3, SENP1, PIAS1, RANBP2, SENP5, and SENP2 were differentially expressed in glioblastoma. A few studies were reported about these genes and consistent with our findings. For example, SENP1 is upregulated in glioblastoma. Knockdown of SENP1 can inhibit the phosphorylation of IκBα and Akt and inhibit the expression of Bcl-xL and cyclinD1, thereby promoting glioblastoma cell apoptosis (Xia et al., 2016). In addition, UBE2I promotes the SUMOylation of CDK6 in glioblastoma and promotes tumor cell development by regulating the cell cycle (Bellail et al., 2014). UBE2I is highly expressed in glioblastoma and promotes the SUMOylation of CRMP2, which in turn drives the proliferation of glioblastoma (Wang and Ji, 2019). Moreover, the upregulated UBE2I can promote the SUMOylation of PUM2 and ultimately promote glioma vasculogenic mimicry (Wang et al., 2020). However, other SUMOylation regulators are rarely reported in glioblastoma. The research on the SUMOylation regulator-related genes provides an important basis and direction for further exploring the research of SUMOylation in the pathogenesis of glioblastoma.

To further analyze the prognostic importance of SUMOylation-related genes, a risk-scoring model of glioblastoma was constructed based on the SUMOylation regulator-related genes. The results of Kaplain–Meier survival curves and ROC curves showed the effectiveness of the model’s prediction. The nomogram based on the risk-scoring model provided a reference for molecular diagnosis, prognosis assessment, and possible targets for patients with glioblastoma.

This work also has some limitations. For example, the expression and function of SUMOylation regulators and their related genes need more basic experimental verification.

## Conclusion

In conclusion, this study analyzed the expression of 15 SUMOylation regulators in glioblastoma. The risk assessment model was constructed based on the SUMOylation regulator-related genes, which had a strong predictive ability for the overall survival of patients with glioblastoma. It might provide targets for the study of the relationship between SUMOylation and glioblastoma.

## Data Availability Statement

Publicly available datasets were analyzed in this study. This data can be found here: Data was acquired from TCGA database (https://cancergenome.nih.gov/), GEO database (https://www.ncbi.nlm.nih.gov/geo/, GSE13041), and cBioportal database (https://www.cbioportal.org/).

## Author Contributions

YM contributed to the experiment design and final inspection. XL contributed to the data analysis and manuscript draft. Both authors have read and approved the final manuscript.

## Conflict of Interest

The authors declare that the research was conducted in the absence of any commercial or financial relationships that could be construed as a potential conflict of interest.

## Footnotes

1 https://cancergenome.nih.gov/

2 https://www.ncbi.nlm.nih.gov/geo/

3 https://www.cbioportal.org/
